# Different transferability of incompatibility (Inc) P-7 plasmid pCAR1 and IncP-1 plasmid pBP136 in stirring liquid conditions

**DOI:** 10.1371/journal.pone.0186248

**Published:** 2017-10-12

**Authors:** Shunsuke Nakazawa, Akira Haramiishi, Kohei Fukuda, Yukie Kanayama, Toshinori Watanabe, Masahiro Yuki, Moriya Ohkuma, Kazuhiro Takeda, Kazuhide Kimbara, Masaki Shintani

**Affiliations:** 1 Applied Chemistry and Biochemical Engineering Course, Department of Engineering, Graduate School of Integrated Science and Technology, Shizuoka University, Hamamatsu, Japan; 2 Department of Bioscience, Graduated School of Science and Technology, Shizuoka University, Hamamatsu, Japan; 3 Biomass Research Platform Team, Biomass Engineering Program Cooperation Division, RIKEN Center for Sustainable Resource Science, Tsukuba, Japan; 4 Japan Collection of Microorganisms, RIKEN BioResource Center, Tsukuba, Japan; University of Parma, ITALY

## Abstract

Self-transmissible plasmids are classified into two types based on their sex pili: short and rigid pili, and long and flexible pili. The transferability of two plasmids with different types of sex pili, pBP136 and pCAR1, was compared in stirring liquid conditions with different cell density. The most probable number method to count transconjugants could detect differences in the transfer frequency with higher resolution in comparison with the conventional CFU counting method. Both plasmids showed higher transfer frequency in high stirring rates than static liquid conditions when the donor and recipient density was 10^6^−10^7^ CFU mL^-1^. The probability of donor-initiated plasmid transfer was investigated by a single-cell-level analysis using a cell sorter. The probability was >36-fold higher for pBP136 than for pCAR1; thus, the simulated transfer frequency of pBP136 was much higher than that of pCAR1 in stirring liquid conditions. Nevertheless, the transfer frequency of pCAR1 was as high as that of pBP136 when the donor and recipient cell density was 10^6^ CFU mL^-1^. This fact indicates that the lower probability of the donor pCAR1 to initiate transfer could be overcome by its high tolerance to the shearing force between donor and recipient cells under higher stirring liquid conditions. Our findings can explain the different survival strategies of these two types of plasmids based on their preferences of transfer conditions.

## Introduction

Conjugative transfer of plasmids is the most important mechanism in horizontal gene transfer due to its high frequency and capability to spread large-sized DNA [1, 2]. For in-depth understanding of plasmid transfer(s) in nature, it is necessary to directly compare their transfer frequency under various conditions. Plasmid transfer is influenced by the type of sex pili, host surroundings (solid surface or liquid environment), cell density, growth rate, host species, nutrient availability, temperature, and high-salt stress [3–10]. The differences in the sex pili of plasmid donors are determined by genes in the transmissible plasmid, and plasmid donors with short and rigid pili prefer solid surfaces to transfer the plasmid, whereas those with long and flexible pili can transfer the plasmid in liquid surroundings [3, 11]. Thus, plasmid survival could be influenced by the differences in environmental conditions between solid surface and liquid environments.

Plasmids with rigid pili can be transferred in liquid conditions; however, their transfer frequency is lower than that on solid surfaces [3, 11]. Notably, their transfer frequency in liquid conditions can still be higher than that of plasmids with flexible pili [3, 11]. In a previous study, we showed the incompatibility (Inc) P-7 plasmid pCAR1 [12–14], which has gene sets encoding flexible type pili [15, 16], was transferred more frequently in liquid conditions than in solid conditions [17–19], especially at lower cell density [10]. Moreover, the transfer frequency of pB10 (IncP-1), which has gene sets encoding short and rigid type pili [20], was higher than that of pCAR1, even in liquid conditions at higher cell density [10]. Thus, rigid-type pili might be more advantageous to plasmids than flexible and long pili, even in liquid conditions. The influence of the flows, streams and movement occurring in natural environments on transferability of plasmids in liquid conditions is poorly documented. In a study that calculated and estimated the transfer frequency in a bacterial population [21], the authors divided the plasmid transfer event into three steps: (i) physical contact between donor and recipient, (ii) initiation of DNA transfer, and (iii) detachment of cells [21]. The conditions in moving liquid conditions are likely to affect the probability of steps (i) and (iii).

The objective of this study was to assess whether the transferability of two plasmids with different types of pili could change in stirring liquid conditions. As model plasmids, IncP-1 plasmid pBP136 [22] with short and rigid type pili [3] and the IncP-7 plasmid pCAR1 were used. The transfer frequency in stirring liquid conditions was determined in different cell density of donor and recipient. The probability of donor-initiated plasmid transfer was also investigated.

## Materials and methods

### Bacterial strains, plasmids, and culture conditions

The bacterial strains used in this study are listed in Table 1. Donor and recipient *Pseudomonas* strains were grown overnight in Luria Broth (LB) [23] at 30°C with appropriate antibiotics. Antibiotics were used at final concentrations of 50 μg/mL for kanamycin (Km), 30 μg/mL for gentamicin (Gm), and 30 μg/mL for rifampicin (Rif) unless otherwise noted. The solid media were prepared by the addition of 1.5% (w/v) agar. Derivatives of plasmids of pBP136 and pCAR1 [pBP136::*gfp* and pCAR1::*gfp* [24]] were used in this study. *Pseudomonas putida* SMDBS (*P*. *putida* KT2440-derivative strain) was used as a donor of these plasmids, and *P*. *putida* KT2440RG (derivative strain of KT2440, spontaneously Rif resistant and Gm resistance gene inserted into chromosome) was used as a recipient.

**Table 1:** Bacterial strains and plasmids used in this study.

Strain or plasmid	Relevant characteristics	Reference
Bacterial strains		
*Pseudomonas putida*		
KT2440RG	Derivative strain of KT2440, spontaneously Rif resistant (Rif^r^), and Gm^r^ gene into chromosome	[25]
SMDBS	A *dapB*-deleted strain of SM1443, Rif^r^ of KT2440 (KT2442) with mini-Tn*5*-*lacI*^q^ cassette inserted into the chromosome (deposited as JCM 31838 in RIKEN BRC-JCM, Japan)	[24]
SMDBS(pBP136::*gfp*)	SMDBS bearing pBP136::*gfp* (JCM 31839)	[24]
SMDBS(pCAR1::*gfp*)	SMDBS bearing pCAR1::*gfp* (JCM 31840)	[24]
Plasmids		
pBP136::*gfp*	pBP136 carrying Km^r^ and P_*A1/O4/O3*_-*gfp* cassette in *parA* (26,137 nt)	[24]
pCAR1::*gfp*	pCAR1 carrying Km^r^ and P_*A1/O4/O3*_-*gfp* cassette in ORF171 (182,625 nt)	[24]

### Mating assays in static and stirring conditions

To evaluate the effect of liquid stirring conditions, 125 mL spinner flasks (Corning Inc., Corning, NY, USA) and a slow speed stirrer (10–1400 rpm, Nisshinrika, Japan) were used. Donor and recipient cells were precultured in 3 mL LB for 12 h at 30°C (140 strokes/min) and then transferred into 200 mL fresh LB with antibiotics in 500 mL flasks (1% seed) and cultured for 15–18 h at 30°C (100 rpm). After the cell densities were adjusted to 10^5^, 10^6^, 10^7^, or 10^8^ CFU/mL, respectively, the cells were harvested at 5,000 × *g*, 4°C, 15 min. The resultant cells were washed with 1/3LB (3.3-g/L of tryptone, 1.7-g/L of yeast extract, and 5.0-g/L of NaCl), and then both were resuspended and mixed with pre-warmed (30°C) and pre-stirring 100 mL 1/3LB. Afterwards, the mixed culture was transferred to the stirring flask. All experiments were performed at least three times. To count colony-forming units (CFUs) of the transconjugants, serial dilutions of mixed samples of donor and recipient strains (10^0^ to 10^8^) were prepared and spread onto selective solid media. For the most probable number (MPN) method to count the transconjugants, serial dilutions of the samples (2^0^ to 2^24^≃10^7.2^) were prepared in a 96-well cell culture plate (Nippon Genetics Co., Ltd. Tokyo, Japan). Afterward, high concentrations of antibiotics (Km; 100 μg mL^-1^, Gm; 60 μg mL^-1^) were added in each well to stop protein syntheses and kill the donor and recipient cells. After incubation of the plate for 2 days at 30°C, the wells in which the transconjugants grew were counted. The resultant data were subjected to calculation of the MPN number and its deviation [26]. Transfer frequency of the plasmids was determined as the number of transconjugants (MPN/mL) divided by the numbers of donor and recipient cells (CFU/mL).

### Evaluation of the probability of donor-initiated plasmid transfer

First, to determine the ratio between donor and recipient cells at which at least one single transfer event occurred in a well, different cell densities of donor (10^0^ to 10^3^) and recipient (10^5^ to 10^7^) were mixed in each well of the 96-well cell culture plates. After 45 min incubation at 30°C, high concentrations of antibiotics (Km; 100 μg/mL, Gm; 60 μg /mL) were added in each well to kill the donor and recipient cells. Then, after incubation of the plate for 2 days 30°C, the wells in which the transconjugants grew were counted. Second, based on the above data, a single donor cell was sorted by cell sorter (FACS, MoFlo XDP IntelliSort II instrument, Beckman Coulter, Denver, MA) into each well filled with 10^7^-CFU/mL recipient cells. The FACS was equipped with a CyClone robotic arm for plate sorting, using a 488-nm argon laser and a 70-μm nozzle orifice. The cell sorting was performed according to the previously described method [24] with slight changes. The sort gate was set based on forward scatter and the side scatter. The validity of the gate was confirmed by sorting single cells each on different points on a plate of solid LB for 384 times and counting how many colonies were formed on the plate out of the sorting cells.

### Simulation of the number of physical cell contact events in different stirring rates

The number of physical contact events between donor and recipient cells in different stirring rates was simulated by collision theory in the chemical reactions. Several assumptions were made: (i) all cells have the same sphere shape, although the *Pseudomonas* strains have a rod shape (ii) cells do not move by themselves, although the *Pseudomonas* strains are motile (iii) a single donor (or recipient) cell can contact a recipient (or donor) cell only one time during the conjugation.

The unit volume of the cells with diameter *d*_*c*_ moving with relative average velocity *u*_*rel*_ per unit of time (1 sec) was $\frac{\pi }{4}$*d*_*c*_^*2*^*u*_*rel*_. The collision frequency (*z*) in the unit volume during a unit time with cell density *N* is shown as (1.1).

$$z=\frac{\pi }{4}{d_{c}}^{2}u_{rel}N$$ (1.1)

The cell distribution in the flask was calculated due to Maxwell distribution (1.2). The velocity vector of the cells was f(*v*_*x*_,*v*_*y*_,*v*_*z*_).

$$f(v_{x},v_{y},v_{z})={\left(\frac{m}{2\pi kT}\right)}^{3/2}exp\left(\frac{-m(v_{x}^{2}+v_{y}^{2}+v_{x}^{2})}{2kT}\right)$$ (1.2)

*m*: mass of cell, *k*: Boltzmann constant, *T*: thermodynamic temperature

Assuming the velocity of the cell as *u*, the *u*_*rel*_ calculated by (1.2) was shown as (1.3).

$$u_{rel}=\sqrt{2}u$$ (1.3)

Total number of contact between the cells *Z* [Hit] at the time (*t*) in the volume of flask (*V*) was calculated with (1.1) and (1.3) as shown in (1.4). Note that the same combination between the two cells were removed (dividing by 2).

$$Z(t)=\frac{\sqrt{2}}{8}\pi {d_{c}}^{2}uN^{2}Vt$$ (1.4)

The spinner flask was cylindrical in shape; thus, the V was shown in (1.5).

$$V=\pi r^{2}H$$ (1.5)

The spinner flask used in this study had a stirring blade with diameter *d* [m], width *b* [m], the number of blades was *n*_*p*_, the diameter of flask was *D* [m], and the height of culture was *H* [m]. The stirring rate was *n* [s^-1^], and the density and viscosity of liquid medium were *ρ* [kg m^-3^] and *μ* [Pa·s], respectively.

Assuming the cell velocity *u* was the same with the velocity of rotational flow *u*_*t*_ under turbulence conditions *u*_*t*_, the *u* was estimated as follows.

$${u=u}_{t}=2\pi nr\;(0\leq r\leq r_{c})$$ (1.6)

$${u=u}_{t}=2\pi nr_{c}{\left(r_{c}/r\right)}^{m1}\;(r_{c}\leq r\leq D/2)$$ (1.7)

*r*_*c*_: rigid-body rotation radius, when the distance from the center of flask (*r*) was equal or smaller than *r*_*c*_, the angular velocity was the same with velocity of rotational flow. When the r was larger than *r*_*c*_, the *m*1 was estimated as 0.8. The *r*_*c*_ was predicted by Nagata’s equation (1.8).

$$\frac{2r_{c}}{d}=1.23\{0.57+0.35(d/D)\}{\left(b/D\right)}^{0.036}n_{p}^{0.116}Re/(10^{3}+1.43Re)$$ (1.8)

The Reynolds number *Re* was defined as equation of 1.9.

$$Re\equiv \frac{nd^{2}\rho }{\mu }$$ (1.9)

The total number of contacts between cells *Z* [Hit] was the sum of the number of cell contacts in two different regions in the flask: one was a rigid-body rotation region *Z1* and the other (*Z2*) was outside of the *Z1* (1.10).

Z(t)=Z1(t)+Z2(t) (1.10)

Z1 was calculated by (1.4), (1.5), (1.6).

$$Z1(t)=\frac{\sqrt{2}}{4}\pi^{3}{d_{c}}^{2}nN^{2}Htr^{3}$$ (1.11)

The equation (1.11) was integrated with respect to *r* from 0 to *r*_*c*_.

$$Z1(t)=\frac{\sqrt{2}}{16}\pi^{3}{d_{c}}^{2}nN^{2}Ht{r_{c}}^{4}$$ (1.12)

Z2 was calculated by (1.4), (1.5), (1.7).

$$Z2(t)=\frac{\sqrt{2}}{4}\pi^{3}{d_{c}}^{2}nN^{2}Ht{r_{c}^{1.8}r}^{1.2}$$ (1.13)

The equation (1.13) was integrated with respect to *r* from *r*_*c*_ to *D*/2.

$$Z2(t)=\frac{\sqrt{2}\pi^{3}{d_{c}}^{2}nN^{2}Htr_{c}^{1.8}({\left\{\frac{D}{2}\right\}}^{2.2}-r_{c}^{2.2})}{8.8(\frac{D}{2}-r_{c})}$$ (1.14)

Therefore, the *Z*(*t*) was calculated by (1.10), (1.12), (1.14).

$$Z(t)=\frac{\sqrt{2}}{16}\pi^{3}{d_{c}}^{2}nN^{2}Ht{r_{c}}^{4}+\frac{\sqrt{2}\pi^{3}{d_{c}}^{2}nN^{2}Htr_{c}^{1.8}({\left\{\frac{D}{2}\right\}}^{2.2}-r_{c}^{2.2})}{8.8(\frac{D}{2}-r_{c})}$$ (1.15)

To remove the contacts between donor to donor, and recipient to recipient, half of *Z*(*t*) was the number of donor and recipient cell contacts. Then the resultant value was divided by the cell density of donor and recipient yielding *Z*_+_. The calculation was performed with the parameters shown in Table 2.

**Table 2:** Parameters used in the simulation of the number of cell-cell contact.

Parameter	Symbol	Unit	Value
Cell density per mL		CFU/mL	4.0×10^6^
Cell density per m^3^	*N*	CFU/m^3^	4.0×10^12^
Diameter of cell	*d*_*c*_	m	1.2×10^−6^
Mass of cell	*m*	kg	7.0×10^−16^
Temperature	*T*	K	3.0×10^2^
Radius of flask	*r*	m	3.1×10^−2^
Height of flask	*H*	m	3.3×10^−2^
Volume of flask	*V*	m^3^	1.0×10^−4^
Diameter of blade	*d*	m	4.0×10^−2^
Diameter of flask	*D*	m	6.2×10^−2^
Width of blade	*b*	m	5.0×10^−2^
Number of blade	*np*	-	2
Density of medium	*ρ*	kg/m^3^	1.0×10^3^
Viscosity of medium	*μ*	Pa∙s	8.0×10^−4^
Duration of time		s	2.7×10^3^

## Results

### MPN method could detect small differences in the frequency of plasmid transfer under different conditions

**Fig 1:**
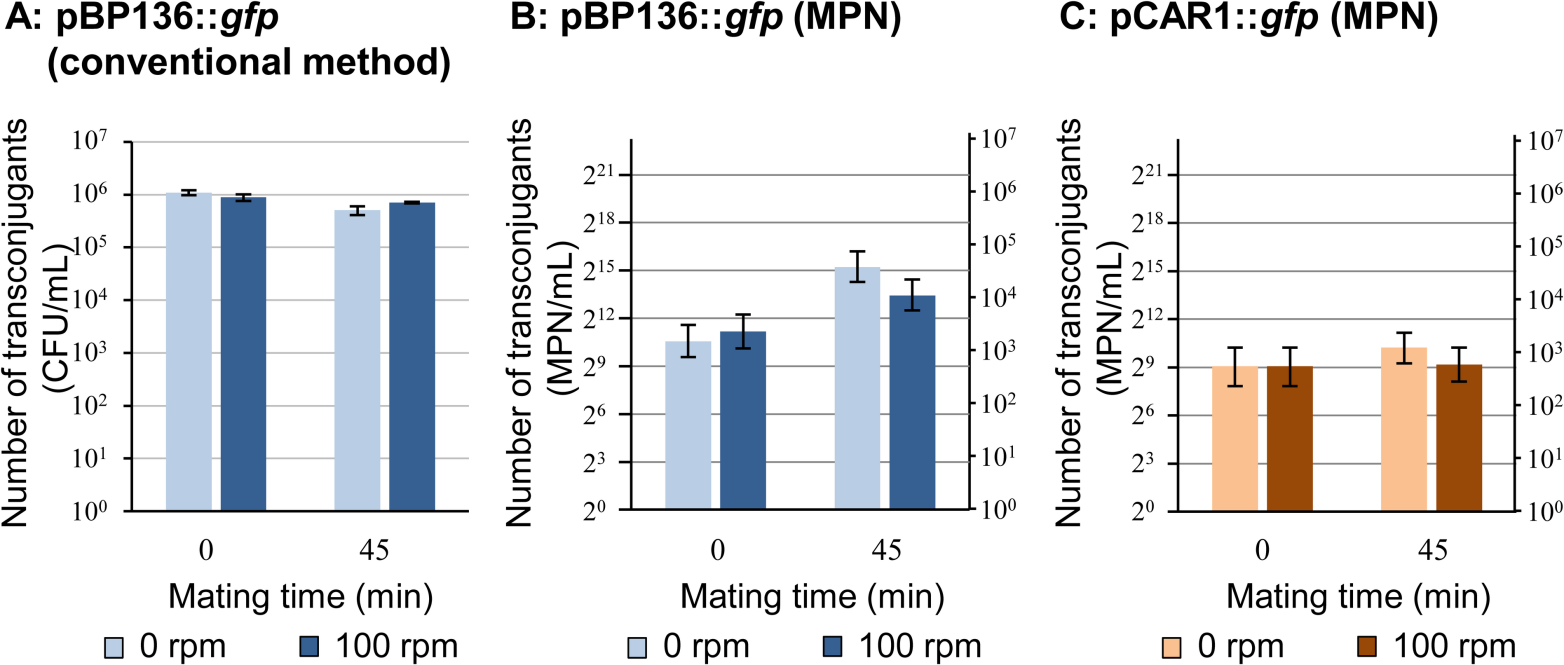
Number of transconjugants of plasmids pBP136::*gfp* and pCAR1::*gfp* under non-stirring (0 rpm) and stirring (100 rpm) conditions. The numbers were counted by the conventional method of counting their CFU (A) and most probable number (MPN) methods (B and C). Because the MPN methods were performed with serial dilutions of the sample (2^0^ to 2^24^≃10^7.2^), two logarithmic scales are shown with bases 2 and 10 (B and C). As a donor, 10^8^ CFU/mL of *P*. *putida* SMDBS was used and *P*. *putida* KT2440RG was used as a recipient. The means of numbers of transconjugants number calculated from triplicate experiments are shown, and standard deviation (A) or 95% confidence limits (B and C) are shown as error bars. The raw data of this figure are in S1 Table.

For the evaluation of the frequency of plasmid transfer, the number of transconjugants was counted by two methods: (i) a conventional method by counting the CFUs of transconjugants on the selective plates, and (ii) counting transconjugants by the MPN method. There were no significant differences in the number of transconjugants with pBP136::*gfp* between non-stirring (0 rpm) and stirring (100 rpm) conditions after 0 min or 45 min conjugation with 10^8^ CFU/mL of donor and recipient strains, respectively (*p*>0.50, Fig 1A). In contrast, the MPN of transconjugants with pBP136::*gfp* under the stirring condition (100 rpm) after 45 min conjugation was significantly smaller than that under the non-stirring condition (Fig 1B, note that the error bar showed 95% confidence limits, respectively). In the case of pCAR1::*gfp*, the differences in the number of transconjugants were less than 4-fold between non-stirring and stirring conditions using the MPN method (Fig 1C, Data Fig 1). The MPN method detected the differences between 0 min and 45 min conjugation with the donors of pBP136::*gfp* (Fig 1B), which could not be distinguished by the CFU-counting-method (Fig 1A).

### Transfer frequency of pBP136::*gfp* and pCAR1::*gfp* changed with different cell density and under different stirring speeds

**Fig 2:**
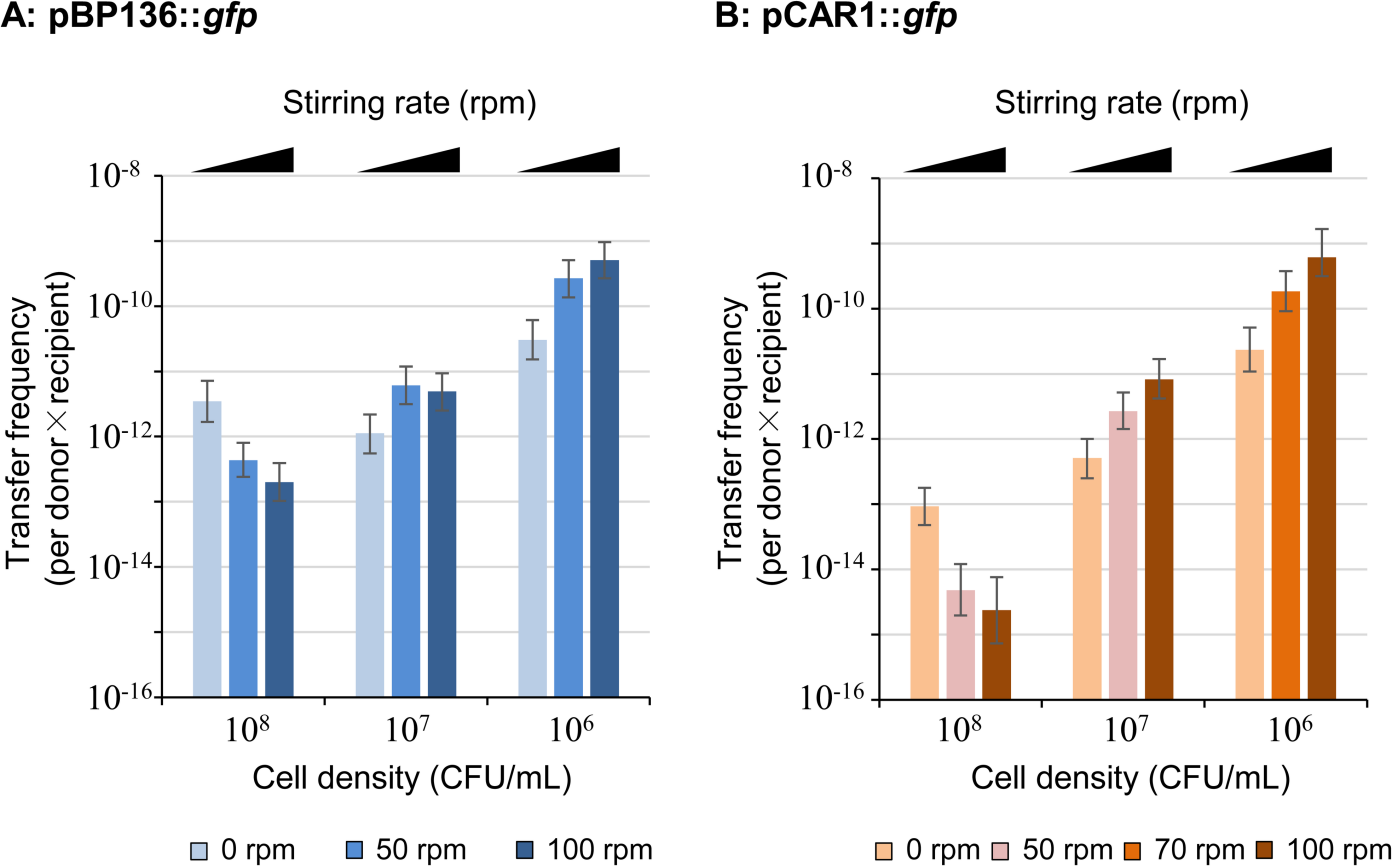
Comparisons of the transfer frequency of pBP136::*gfp* (A) or pCAR1::*gfp* (B) with different cell densities, 10^8^, 10^7^, and 10^6^ CFU/mL in different stirring conditions (0, 50 or 70, and 100 rpm). The raw data of this figure are in S2 Table.

**Fig 3:**
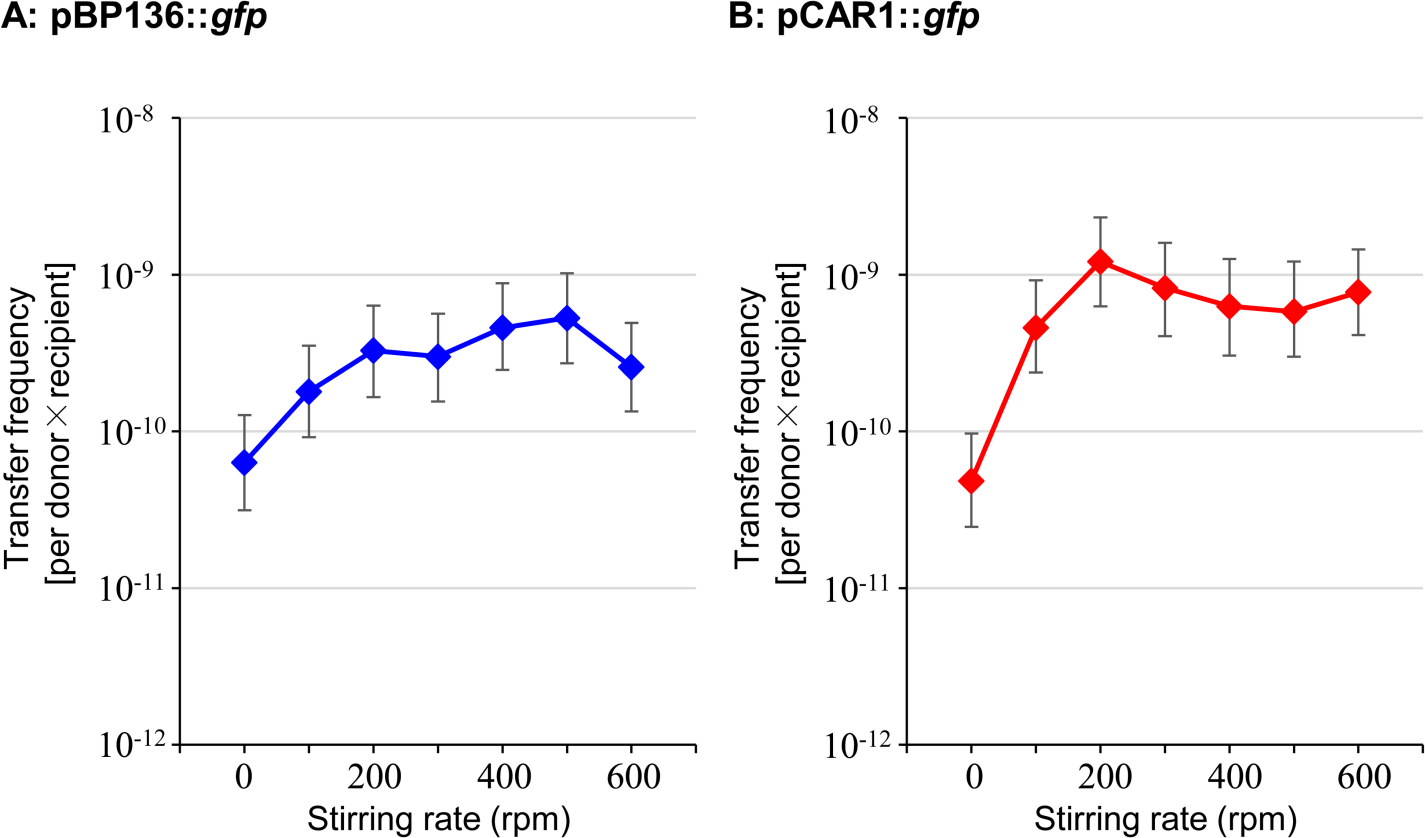
Comparisons of the transfer frequency of pBP136::*gfp* (A) or pCAR1::*gfp* (B) at cell density of 10^6^ CFU/mL in higher stirring conditions (0 to 600 rpm). The raw data of this figure are in S3 Table.

Comparisons of the number of transconjugants harboring pBP136::*gfp* or pCAR1::*gfp* by the above MPN methods were performed with different cell densities, 10^8^, 10^7^, and 10^6^ CFU/mL at non-stirring conditions, and then the transfer frequency was calculated for each condition. As shown in Fig 2, the transfer frequency of both plasmids with 10^8^ CFU/mL cell density decreased in the higher stirring conditions (50 and 100 rpm). Their transfer frequency was higher in pBP136::*gfp* than that in pCAR1::*gfp* (Fig 2). In contrast, when the cell density was 10^7^ or 10^6^ CFU/mL, the transfer frequency increased in higher stirring rate conditions (Fig 2). Notably, with 10^6^ CFU/mL cell density and 100 rpm stirring rate condition, the transfer frequency of pCAR1::*gfp* (≃6.2×10^−10^, Fig 2B) was equal to or higher than that of pBP136::*gfp* (≃5.0×10^−10^, Fig 2A). Because the number of transconjugants of both plasmids increased in high stirring rates with 10^6^ CFU/mL cell density, mating assays were performed in higher stirring rate conditions (up to 600 rpm). As a result, the transfer frequency increased under higher stirring rate in both cases of pBP136::*gfp* and pCAR1::*gfp* (Fig 3). The differences in the frequency of pCAR1::*gfp* transfer were ~ 25-fold (Fig 3B), whereas those of pBP136::*gfp* were less than 10-fold (Fig 3A).

### Probability of donor-initiated plasmid transfer

The transfer frequency was also affected by how often the donor initiated the plasmid transfer. In our detection system of transconjugants using a 96-well plate, a single plasmid transfer event in each well could be counted because only the transconjugant could survive in the culture in the well after the conjugation. To determine the ratio of donor and recipient cells in which at least one single transfer event occurred, mating assays with different densities of donor and recipients were performed. Thus, transconjugants of pBP136::*gfp* were detected in 100% of 96-mating pairs when the cell densities were 10^3^ CFU/mL of donor with 10^5^−10^7^ CFU/mL of recipient, and 10^2^ CFU/ mL of donor with 10^6^−10^7^ CFU/mL of recipient (Table 3). When 10^2^ CFU/mL of donor was mated with 10^5^ CFU/mL of recipient, the number of wells with transconjugants decreased (56%, Table 3). When 10^1^ CFU/mL of donor and 10^5^ CFU/mL of recipient were mated, the percentage of wells with transconjugants drastically decreased (2.1%, Table 3). In the case of pCAR1::*gfp*, the percentage of wells with transconjugants was lower than that of pBP136::*gfp* (Table 3), indicating that the probability of donor-initiated transfer of pCAR1::*gfp* was lower than that of pBP136::*gfp*. Based on these results, single donor cells were each sorted by FACS into a well filled with 10^7^ CFU/mL of recipient, and the numbers of wells with transconjugants were counted. Thus, the number of wells with transconjugants of pBP136::*gfp* was larger (1.9%) than that of pCAR1::*gfp* (<0.052%) (Table 4). The average validity of the sorting was 90.9–96.4% for donor of pBP136::*gfp* and 86.2–95.3% for donor of pCAR1::*gfp*, and the above values were recognized as the initiation probability of each plasmid, *Pt*_pBP136_ = 1.9×10^−2^, and *Pt*_pCAR1_ = 5.2×10^−4^.

**Table 3:** The number of wells with transconjugants detected in the mating assays with different cell density.

Plasmid	^a^Donor [CFU/well]	^a^Recipient [CFU/well]	The numbers of wells with transconjugants [wells]	Percentage [%]
pBP136::*gfp*	10^3^	10^7^	96	100
		10^6^	96	100
		10^5^	96	100
	10^2^	10^7^	96	100
		10^6^	96	100
		10^5^	54	56
	10^1^	10^7^	71	74
		10^6^	63	66
		10^5^	2	2.1
pCAR1::*gfp*	10^2^	10^7^	6	6.3
		10^6^	6	6.3
		10^5^	0	0
	10^1^	10^7^	1	1.0
		10^6^	1	1.0
		10^5^	0	0
	10^0^	10^7^	0	0
		10^6^	0	0
		10^5^	0	0

^a^The numbers of donor and recipient cells were only counted in the samples with the highest cell density. Donor of pBP136::*gfp* or pCAR1::*gfp* was 4.9×10^3^ and 1.8×10^2^, respectively, while the recipients of them were 3.2×10^7^ and 5.3×10^7^, respectively.

**Table 4:** The number of wells with transconjugants detected in the mating assays at a single cell level.

Plasmid	Cell number of the donor [cell/well]	Recipient [CFU/well]	Total well numbers [well]	The numbers of wells with transconjugants [well]	Percentage [%]
pBP136::*gfp*	1	10^7^	1212	23	1.9
pCAR1::*gfp*	1	10^7^	1920	1	<0.052

### Simulated transfer frequency of plasmids in different stirring rates

**Fig 4:**
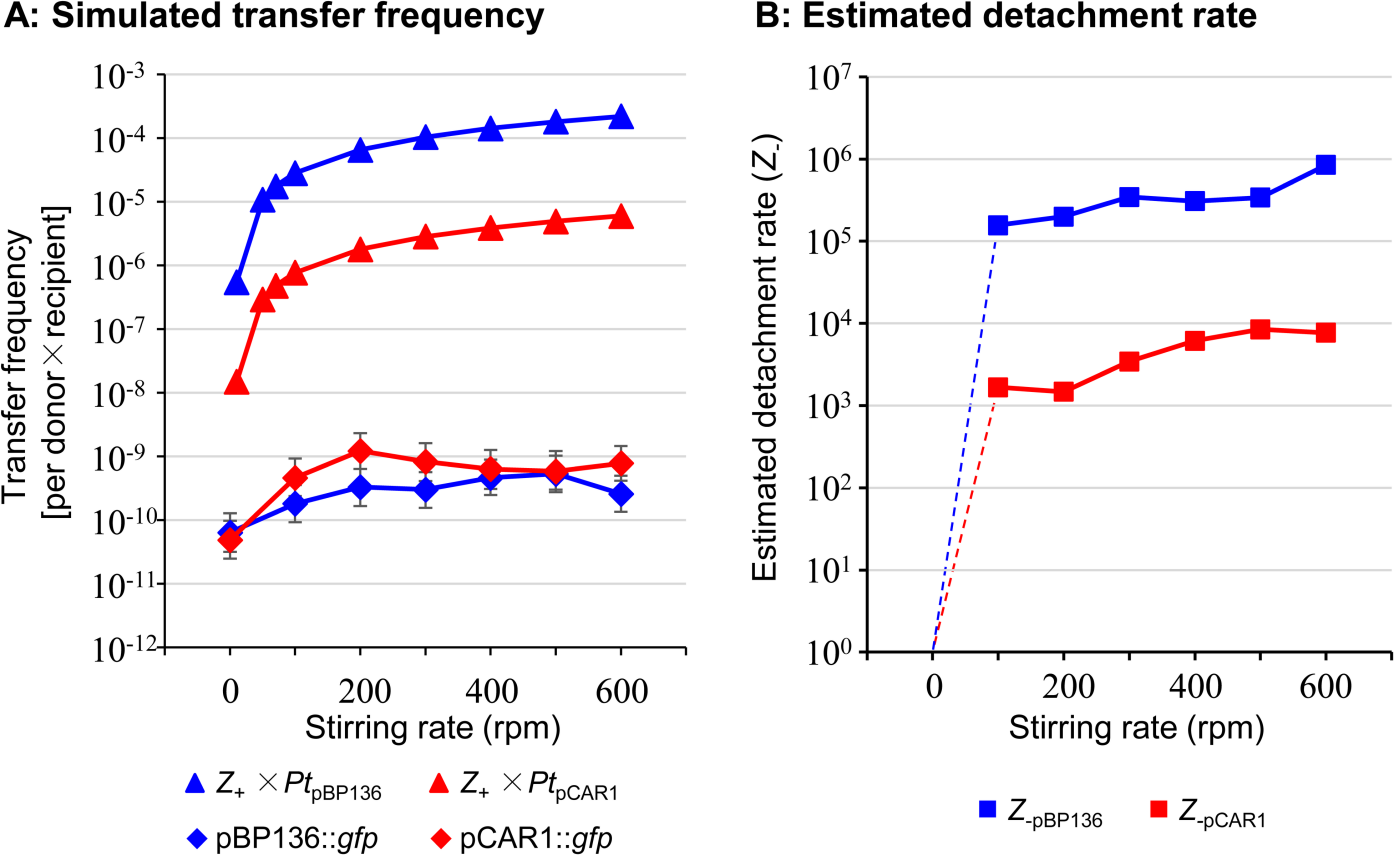
The simulated transfer frequency and detachment rates of plasmids. (A) The simulated and experimental transfer frequency of pBP136::*gfp* and pCAR1::*gfp* in different stirring rates at cell density of 10^6^ CFU/mL. Note that the simulated transfer frequency are shown from 10 to 600 rpm. The experimental data of the number of transconjugants shown in Fig 3 are also shown in the graph for comparison, blue (pBP136::*gfp*) and red (pCAR1::*gfp*). (B) The estimated detachment rates (*Z*_-_) in different stirring rates. Blue; *Z*_-pBP136_ and red; *Z*_-pCAR1_. The detachment rate at 0 rpm could not be calculated but be closer to 0 (dash lines). The raw data of this figure are in S4 Table.

The simulated transfer frequencies of the two plasmids were calculated with *Z*_+_ and *Pt* of each plasmid from 10 to 600 rpm as shown in Fig 4A. The simulated transfer frequency was much higher than that of experimental data for each plasmid: 10^3^−10^4^ -fold for pBP136::*gfp* and 10^2^−10^3^ -fold for pCAR1::*gfp* (Fig 4A).

## Discussion

Our findings in the present study for the transferability of two plasmids pBP136 and pCAR1 with different types of pili in stirring liquid conditions are very important to understand how the plasmids were spread in natural environments.

We introduced an MPN counting method to count transconjugants and evaluate transfer frequency. Although the MPN-counting method is no more accurate and precise than a direct counting procedure using microscopy [27], this method has been used for the detection and counting of organisms in soils or sediments, which were masked by soil particles and difficult to be counted by microscopy [28, 29]. A conventional method for counting the CFUs of transconjugants on selective plates resulted in a large background because donor and recipient cells at high density could mate on the selective plates during incubation to detect the colonies. For the MPN method, the presence of standard concentration of antibiotics (Km and Gm) might have higher effects on stopping protein synthesis and killing the donor and recipient cells in the well than the conventional method, because the background was reduced (data not shown). By adding the high-concentration antibiotics to the wells, conjugation does not occur in the well during the detection. The background of the plasmid transfer was successfully reduced by introducing this method (Fig 1). The number of transconjugants of two kinds of plasmids detected by the MPN method (10^3^−10^4^ MPN mL^-1^ Fig 1B and 1C) may be due to background since some plasmids could be transferred in the mixing step of donor and recipient cells within a few minutes [30, 31]. The estimated backgrounds of transfer frequency in the case of 10^8^ CFU/mL cell density were 10^−14^–10^−13^ for pBP136::*gfp* and pCAR1::*gfp*. The backgrounds in different cell density were 10^−13^–10^−12^ for both plasmids (10^7^ CFU/mL) and 10^−12^–10^−11^ for pBP136::*gfp* (10^6^ CFU/mL). The transconjugants of pCAR1::*gfp* in the case of cell density of10^6^ CFU/mL were under detection limit, thus the background transfer frequency might be below 10^−13^.

As for the duration of conjugation, 45 min was selected for two reasons: (i) the doubling time of donor and recipient cells in LB was 1.5–2.0 h (data not shown), and therefore, the duration was set to less than the doubling time to exclude the possibility of the increase of transconjugants by cell division. (ii) no or few transconjugants were detected in shorter durations (<45 min) at 0 rpm condition with cell density at 10^6^ CFU/mL (for 0 min conjugation of pBP136::*gfp*: 4–200 MPN/mL, 10 min: 48~360 MPN/mL, 30 min: 170~780 MPN/mL, 45 min: 300–3500 MPN/mL). Longer DNA might take a longer time to transfer, and the sizes of plasmids in this study were 44 kb (pBP136::*gfp*) and 200 kb (pCAR1::*gfp*). It is therefore possible that the transfer of pCAR1::*gfp* takes 4 times longer than pBP136::*gfp*. The differences in size of these plasmids might affect the results of plasmid transfer, especially in higher stirring rate conditions. This possibility could be tested by using plasmids with the same type of pili but with different sizes. As for the stability of them, both could be stably maintained in the presence of Km (data not shown), even though the *gfp* gene were inserted into *parA* (involved in multimer resolution system) on pBP136. Thus, the stability of plasmids were not the factors for the differences of transfer frequency in the present study.

Notably, the transfer frequency of both plasmids increased in lower cell density even in static conditions (0 rpm) (Fig 2), which was similar to the results of our previous report [10]. The reason for this is unclear, but it is possible that several numbers of donor cells might not be paired with recipient cells, and/or some donor and recipient pairs could not initiate plasmid transfer. It should be noted that higher stirring rate increased the transfer frequency in the cases of lower cell density but not in high cell density (Fig 2). The higher stirring rate increased not only the number of the contact events but also the shearing force between cells, which caused cell-cell detachment [21]. Therefore, the higher stirring rate condition was a compromise between increasing the number of cell-to-cell contact events and increasing the cell-cell detachment. If the contact events of donor and recipient were relatively high in the static condition (0 rpm), the increase in the shear force with higher stirring rate conditions might reduce the transfer events of plasmids. This was likely the major reason why the transfer frequency of both plasmids decreased with 10^8^ CFU/mL density (Fig 2A and 2B). In the case of lower cell density, stirring could increase the number of contact events resulting in higher transfer frequency.

The transfer frequency was also determined by how often the donor initiated the plasmid transfer. The estimated probability of the initiation was >36-fold higher in pBP136::*gfp* than that in pCAR1::*gfp* (Table 4), which coincided with the static liquid condition at 10^8^ CFU/mL cell density (Fig 2). However, this difference was larger than the detected differences in the mating assays in stirring liquid conditions at lower cell density (10^6^−10^7^ CFU/mL) (Figs 2 and 3). IncP-7 plasmids, including pCAR1, are predicted to have long and flexible-type pili based on the nucleotide sequences of the genes (*tra*/*trh*) [12, 15, 16], and this plasmid may be preferentially transferred in liquid conditions and be tolerant to the stirring conditions [3]. In contrast, IncP-1 plasmids, including pBP136, have genes for short and rigid-type pili [15, 16] and they are considered to be preferentially transferred on solid surface conditions [3]. In fact, these preferences of IncP-7 and IncP-1 plasmid transfers were found in our previous report [10].

Despite considering the probability of donor-initiated plasmid transfer (*Pt*), the simulated transfer frequency of each plasmid was still much larger than the experimental data (Fig 4A). It should be also noted that the simulated frequency of pCAR1::*gfp* was lower than that of pBP136::*gfp* (Fig 4A), because *Pt*_pCAR1_ was much lower (36-fold) than *Pt*_pBP136_. Nevertheless, the experimentally determined transfer frequency of pCAR1::*gfp* in the higher stirring conditions was as high as that of pBP136::*gfp* (Fig 4A). This was because the negative effect of stirring (shearing force) was not taken account in the simulation shown in Fig 4. Zhong et al. calculated the attachment and detachment rates for each shaking speed using experimental data [21]. In this study, we estimated the detachment rate (*Z*_-_) of the donor and recipient cells in different stirring conditions using experimental data and the simulated Z_+_. Here, the *Z*_-_ value was defined as 1 when the stirring rate is 0. Therefore, the transfer frequency of the plasmid (*F*) could be estimated as (1.16).

$$F=Pt\frac{Z_{+}}{Z_{-}}$$ (1.16)

The detachment rate *Z*_-_ under the stirring conditions was estimated as shown in Fig 4B, which was different between pBP136::*gfp* and pCAR1::*gfp*. Both *Z*_-_ values increased at higher stirring rate of liquid, and the detachment rates reached stable values between 100–600 rpm (Fig 4B). The reason why the *Z*_-pBP136_ was ~ 100-fold higher than *Z*_-pCAR1_ was probably the differences in the pili types of their donors. Notably, the previous report by Zhong et al. also showed the estimated detachment rates of plasmids pB10 (also belongs to IncP-1, encoded rigid type pili), F´, and R1 (both plasmids encoded flexible type pili) [21]. The detachment rate of pB10 was 10–25 fold higher than those of the others [21]. Their estimation was based on the numbers of transconjugants obtained by conventional counting methods, and thus it is possible that the differences between the plasmids with different types of pili were underestimated. Our estimation indicated that the long and flexible type pili encoded on pCAR1::*gfp* was 100-fold resistant to that on pBP136::*gfp*. Even though there is a deleterious factor on plasmid transfer by stirring, donors of pCAR1::*gfp* might be more tolerant to the stirring liquid conditions than those of pBP136::*gfp*. This higher tolerance of the flexible pili in the donor of pCAR1 could allow them to overcome the lower transfer frequency. Clarke et al. showed the long and flexible pili encoded on F plasmid could help the donor and recipient cells survive in liquid with high shear forces [32]. It is therefore possible that the donor of pCAR1::*gfp* encoding long and flexible pili can withstand high shearing force than that of pBP136::*gfp*. In the static liquid conditions (0 rpm), the transfer frequency of the two plasmids were also similar, although the simulated frequency could be different at lower stirring speed (10 rpm in Fig 4A). We could not clearly explain the reason but the cell movements with different sex pili could affect the cell-cell contact in the static condition, which was ignored in our simulation. It is also possible that the effect of cell density could be larger on the transfer frequency in the static conditions.

Since many habitats of bacteria have liquid flows and movements, including natural environments (soil, river, ocean, animal gut) and/or artificial environments (wastewater treatment plant, air filter, fermentation plant), the estimation of how often a plasmid is transferred in these settings is important to prevent unintended spread of genes or to develop new technologies for controlling these environments. This study clearly showed that the plasmid encoding long and flexible pili could be highly spread, especially under stirring liquid conditions. Our findings also indicate that transfer frequency of such plasmids might be underestimated if the assessment is based only on mating assays in static conditions.

## Conclusion

Many new plasmids have been found in genomic and metagenomics analyses as recently reviewed [16]. For the simulation and prediction of the transferability of these new plasmids, it is important to consider the flow of liquid in their surroundings and features of their sex pili. Recently, in-depth analyses of the behaviors of microbes in flow have been reported not only based on mathematical simulation, but also experimental data using emerging technologies such as real-time visualization [33]. Comparison of the transferability of plasmids in fluid flow conditions with these visualization methods will also be very important to understanding *in situ* spreading of plasmids in natural environments.

## Supporting information

S1 Table Raw data shown in Fig 1. (XLSX)

S2 Table Raw data shown in Fig 2. (XLSX)

S3 Table Raw data shown in Fig 3. (XLSX)

S4 Table Raw data shown in Fig 4. (XLSX)
