# Correction: Different transferability of incompatibility (Inc) P-7 plasmid pCAR1 and IncP-1 plasmid pBP136 in stirring liquid conditions

**DOI:** 10.1371/journal.pone.0191393

**Published:** 2018-01-11

**Authors:** Shunsuke Nakazawa, Akira Haramiishi, Kohei Fukuda, Yukie Kanayama, Toshinori Watanabe, Masahiro Yuki, Moriya Ohkuma, Kazuhiro Takeda, Kazuhide Kimbara, Masaki Shintani

The name of the recipient strain *Pseudomonas putida* KT2440RG appears incorrectly throughout the article. The correct strain is *Pseudomonas putida* KT2440RGD.

There is an error in reference 25. The correct reference is: Shintani M, Li MY, Matsui K, Ohkuma M, Okada K, Nojiri H. (2012) Comparisons of conjugation frequency in different environmental conditions. J Environ Biotechnol 12, 163–167 (Japanese).
